# Retrospective Analysis of Surgical Site Infection After Titanium Plate Removal Following Orthognathic Surgery

**DOI:** 10.3390/jcm14113657

**Published:** 2025-05-23

**Authors:** Kazuyuki Yusa, Tomoharu Hemmi, Satoshi Kasuya, Nobuyuki Sasahara, Shigeo Ishikawa

**Affiliations:** Department of Dentistry, Oral and Maxillofacial-Plastic and Reconstructive Surgery, School of Medicine, Yamagata University, Yamagata 990-9585, Japan; henmi@med.id.yamagata-u.ac.jp (T.H.); silversoarer0315@gmail.com (S.K.); sasanobu8006@yahoo.co.jp (N.S.); shigeo_ishikawa2011@yahoo.co.jp (S.I.)

**Keywords:** orthognathic surgery, plate removal, surgical site infection

## Abstract

**Background/Objectives:** This study aimed to investigate associations between surgical site infection (SSI) and plate removal following orthognathic surgery. **Methods:** The study sample consisted of 191 patients (126 females, 65 males). Plate removal was performed in 174 patients with a mean age of 26.4 ± 9.7 years. Multiple logistic regression analysis was used to estimate odds ratios (ORs) and 95% confidence intervals (CIs) for risk factors for SSI after plate removal. **Results:** Forty-three patients developed SSI after plate removal. The only risk factor independently associated with SSI after plate removal was a history of SSI after orthognathic surgery (OR 2.476, 95% CI 1.040–5.892). **Conclusions:** Patients who experience SSI after orthognathic surgery are at higher risk of SSI after plate removal, so protocols for perioperative management should be carefully considered.

## 1. Introduction

Titanium miniplate fixation has been widely applied for maxillofacial fracture and orthognathic surgery due to the biocompatibility and strength of the plates [1]. Although orthognathic surgery, performed on the maxilla and mandible to correct facial deformities and improve occlusal function, is commonly considered a safe and effective procedure. However, like any major surgery, it carries the potential for complications. Common complications reported in the previous reports include neurosensory disturbance, soft tissue and/or periodontal injuries, postoperative malocclusion, and temporomandibular joint disorder [2,3]. These complications can sometimes require additional treatment, highlighting the complexity of achieving optimal long-term outcomes. And issues such as bone healing and delayed recovery may also affect treatment results, potentially impacting both esthetics and functional recovery. Further, surgical site infection (SSI) is a typical complication of orthognathic surgery. SSI can lead to additional medical interventions, and many reports have investigated the relationship between SSI and orthognathic surgery [4]. In addition to SSI, there are other complications, such as fixation material failures, suture dehiscence, and complications related to the fixation plates themselves. Failures in fixation materials may necessitate the removal of the plates, contributing to further complications.

The removal of the titanium plate is a notable issue in orthognathic surgery management. At present, standardized guidelines or protocols for plate removal are lacking, which may be why the incidence of plate removal after orthognathic surgery has been reported to vary widely, from 3.2% to 27.5%, in studies across several countries [1,5,6,7,8].

While the complications of orthognathic surgery have been extensively reported, relatively few studies have examined postoperative complications (including SSI) of plate removal. The aim of this study was thus to investigate associations between SSI and plate removal following orthognathic surgery. By examining this association, we hope to gain a clearer understanding of the risks involved in titanium plate removal and improve patient management strategies to minimize complications.

## 2. Materials and Methods

### 2.1. Patients

This retrospective study included patients who underwent orthognathic surgery in the Department of Dentistry, Oral and Maxillofacial Surgery at Yamagata University Hospital between 2013 and 2022. Orthognathic surgeries included Le Fort I osteotomy (LF1), bilateral sagittal split osteotomy (BSSO) and genioplasty. Patient age, sex, surgery procedure (LF1, BSSO, or genioplasty), and whether plate removal was performed were reviewed.

### 2.2. Inclusion and Exclusion Criteria

Eligible participants had skeletal dentofacial deformities with prognathism, retrognathia, open bite, and facial asymmetry. Patients with incomplete data, such as discontinuation of medical visits, were excluded in this study. Furthermore, cases in which wisdom tooth extraction or other surgery had been performed at the same time as plate removal were also excluded.

### 2.3. Plate Removal

All patients underwent orthognathic surgery (LF1/BSSO/genioplasty) and were regularly followed by their attending physicians, who confirmed their desire for plate removal after X-ray and CT findings confirmed bone healing. Plate removal was performed according to the customary procedure under general anesthesia with intraoral access through a mucosa incision. A full-thickness mucoperiosteal flap was reflected. The plate was clearly identified and removed. If the plate was covered by bone, the bone was appropriately removed. The wounds were sutured with polyglactin (Vicryl) in two layers. All surgeries were performed either by board-certified surgeons or under their supervision.

### 2.4. Evaluation of Surgical Site Infection After Plate Removal Following Orthognathic Surgery

Clinical evaluations were conducted for those patients who underwent plate removal. The presence of SSI was defined by the presence of purulent discharge and/or purulent collection, or secondary appearance of edema, wound dehiscence, or local inflammatory signs as diagnosed by the attending physician. Patient age, sex, surgical procedure (LF1, BSSO or genioplasty), interval between orthognathic surgery and plate removal, and serum albumin concentration and duration of antibiotic prophylaxis after plate removal were reviewed retrospectively.

### 2.5. Statistical Analyses

A Mann–Whitney U test and chi-squared test were performed to investigate associations between the appearance of SSI after plate removal and clinical variables. To assess whether preoperative conditions correlated with SSI after plate removal, multiple logistic regression analysis was also performed. To reveal the independence of factors related to SSI after plate removal, multiple logistic regression (forced entry) analysis was performed using those variables showing values of *p* < 0.15 in univariate analyses. Statistical significance was set at the level of *p* < 0.05.

### 2.6. Ethical Considerations

All procedures performed in studies involving human participants were in accordance with the ethical standards of the institutional and/or national research committee and the Declaration of Helsinki and its later amendments or comparable ethical standards. Approval was obtained from the ethics committee at Yamagata University Faculty of Medicine (2018-28 and 2024-333: date of approval: 26 April 2018 and 25 March 2025). This retrospective observational study was undertaken using the opt-out method of assuming consent via our hospital website.

## 3. Results

The study sample comprised 191 patients, including 126 females (66.0%) and 65 males (34.0%). Surgeries performed included LF1, BSSO, and genioplasty, either alone or in combination. LF1 was performed in 120 cases, BSSO in 184 cases, and genioplasty in 10 cases. Plate removal was carried out in 174 cases (91.1%), while the remaining 17 patients (8.9%) did not request plate removal (Table 1). Patients who underwent plate removal had a mean age of 26.4 ± 9.7 years (range, 17–70 years), with 114 females (65.5%) and 60 males (34.5%) (Table 1). Among these patients, LF1 was performed in 118 cases, BSSO in 170 cases, and genioplasty in 8 cases. The mean interval between orthognathic surgery and plate removal was 12.9 ± 4.7 months (range, 5–35 months). Table 2 shows the distribution of SSIs after plate removal. Forty-three cases (26 females, 17 males, mean age 25.8 ± 8.6 years) were diagnosed with SSI after plate removal. The chi-squared test revealed significant differences in the distribution of SSIs after orthognathic surgery (*p* = 0.006), with a significantly higher frequency of SSI among patients who had experienced SSI following orthognathic surgery (before plate removal). No statistically significant differences were found for age (*p* = 0.99), sex (*p* = 0.226), or the duration from orthognathic surgery to plate removal (*p* = 0.563).

Furthermore, the only risk factor independently associated with SSI after plate removal was the occurrence of SSI following orthognathic surgery (OR 2.476, 95% CI 1.040–5.892) (Table 3).

## 4. Discussion

The present study aimed to evaluate factors associated with SSI after plate removal. Postoperative SSI as a complication of orthognathic surgery was identified as a possible risk factor for the development SSI after plate removal. In recent years, resorbable plates have been widely used in orthognathic surgery [9]. However, there is currently no clear evidence of a difference between titanium and resorbable plates in terms of postoperative pain and discomfort, patient satisfaction, plate exposure, or infection. As a result, titanium plates continue to be widely used [10]. Some reports have indicated that plate removal is not routinely performed but is undertaken in response to identification of SSI, exposure of the titanium plate, reports of pain around the plating area, or at the request of the patient. Previous reports on plate removal have involved relatively small cohorts [11,12,13], so information on complications after plate removal has been lacking, particularly with regard to risk factors for SSI. It has also been reported that albumin levels may be a risk factor for the development of SSI in various surgical procedures [14,15]. Shigeishi et al. reported that serum albumin levels, as well as operative time and blood loss, are associated with the development of postoperative SSIs in oral surgery, including orthognathic surgery [16]. On the other hand, serum albumin levels were not a risk factor for the development of SSI of plate removal. This may be due to the relatively early resumption of oral intake in plate removal.

According to a report based on a national survey in 2017 by the Japan Society for Jaw Deformities, nearly 70% of Japanese facilities perform plate removal for all (or in principle all) patients after orthognathic surgery [17]. The reason for this has been suggested to be that although the biosafety of titanium plates is understood, resistance to residual foreign material may remain an issue. The data on plate removal presented in this study may thus be important for improving perioperative management in hospitals where plate removal is routinely performed, as well as in hospitals where it is not.

In cases of orthognathic surgery, many reports have indicated that the duration of antibiotic prophylaxis may represent a risk factor for the development of SSI. Although this point remains controversial, some reports have suggested that longer antibiotic prophylaxis for SSI may be effective in some cases [18,19]. Interestingly, the present results showed no significant difference in the duration of antibiotic prophylaxis between cases with or without SSI after plate removal. On the other hand, a history of SSI after orthognathic surgery was associated with a significantly higher risk of SSI after plate removal (Table 2 and Table 3). Although plate removal in this study was performed as usual regardless of case background, plate removal in patients with previous postoperative SSI after orthognathic surgery may need to be addressed as a surgical procedure treating a dirty or infected region. Although not common in plate removal, various methods of drainage have been reported in attempts to reduce SSI for surgeries in the oral and maxillofacial region [20,21]. Such methods may be important in plate removal where the risk of SSI developing is high. Further, as mentioned earlier, the optimal duration of antibiotic prophylaxis for SSI after orthognathic surgery remains contentious. In this study, the duration of antibiotic prophylaxis was not a risk factor for the development of SSI after plate removal, but this may have been influenced by the relatively short duration of antibiotic prophylaxis in both the SSI (mean, 1.8 ± 1.4 days) and non-SSI (mean, 2.3 ± 1.8 days) groups after plate removal. Sahni mentioned that in patients undergoing orthognathic surgery, long-term (2–7 days) antibiotic prophylaxis reduces the risk of SSI when compared to antibiotic administration for only a single day [19]. In the future, the duration of antibiotic prophylaxis for both plate removal and orthognathic surgery may need to be considered.

This study had several limitations. First, eligible cases were not classified according to the surgical procedure applied as orthognathic surgery. The possibility that the incidence of SSI after plate removal may vary depending on preceding surgical techniques should be considered. Future studies should incorporate subgroup analyses according to the type of surgical technique used. A second limitation was that this study used a retrospective design, and prospective studies will be needed in the future. Third, other risk factors for SSI after plate removal may exist, so further studies of larger numbers of participants are needed to confirm our findings. Fourth, a formal statistical power analysis was not conducted prior to the study. The sample size was determined based on the available patient data during the study period. Although the total number of patients (*n* = 191) was deemed adequate for the primary analyses, the statistical power for subgroup analyses may have been limited. Therefore, the results of the subgroup analyses should be interpreted with caution.

## 5. Conclusions

Even in hospitals where plate removal following orthognathic surgery is not routinely performed, SSI after orthognathic surgery is likely to be the major cause of plate removal. The present results suggest that such patients may have an increased risk of infection even after plate removal and that protocols for perioperative management should be carefully considered.

## Figures and Tables

**Table 1:** Characteristics of the patients.

Plate Removal	+	−
	174	17
Age (years); mean ± SD	26.4 ± 9.7	-
Sex		
Male	60	5
Female	114	12
Surgery		
Le Fort I	118	2
BSSO	170	14
Genioplasty	8	2
Orthognathic surgery to plate removal (months); mean ± SD	12.9 ± 4.7	-
Serum albumin (g/dL)	4.6 ± 0.3	-
Duration of antibiotic prophylaxis (days)	2.6 ± 1.7	-

BSSO, bilateral sagittal split osteotomy; SD, standard deviation.

**Table 2:** Quantitative characteristics of participants according to the presence or absence of SSI after plate removal.

			*p*-Value
**SSI After Plate Removal**	**+**	**−**	
	43	131	
Age (years)	25.8 ± 8.6	26.5 ± 10.0	0.999
Sex			0.226
Male	17	43	
Female	26	88	
SSI after orthognathic surgery			0.006
**+**	13	16	
**−**	30	115	
Orthognathic surgery to plate removal (months)	12.7 ± 4.6	12.9 ± 4.7	0.563
Serum albumin (g/dL)	4.6 ± 0.3	4.5 ± 0.3	0.059
Duration of antibiotic prophylaxis (days)	1.8 ± 1.4	2.3 ± 1.8	0.112

SSI, surgical site infection.

**Table 3:** Adjusted ORs and 95% CIs for variables associated with SSI after plate removal.

	SSI After Titanium Plate Removal		
	OR	(95% CI)	*p*-Value
SSI after orthognathic surgery	2.476	(1.040–5.892)	0.040
Serum albumin	3.200	(0.916–11.183)	0.068
Duration of antibiotic prophylaxis	0.816	(0.640–1.040)	0.100

CI, confidence interval; OR, odds ratio; SSI, surgical site infection.

## Data Availability

The data are not publicly available due to privacy concerns but can be accessed upon reasonable request from the corresponding author.

## Abbreviations

The following abbreviations are used in this manuscript:

SSI	Surgical site infection
ORs	Odds Ratios
CIs	Confidence Intervals
LF1	Le Fort I osteotomy
BSSO	Bilateral Sagittal Split Osteotomy
